# 3,4,5-Trimeth­oxy-*N*-(2-methoxy­phen­yl)benzamide

**DOI:** 10.1107/S1600536809027974

**Published:** 2009-07-22

**Authors:** Aamer Saeed, Ulrich Flörke

**Affiliations:** a Department of Chemistry, Quaid-i-Azam University, Islamabad 45320, Pakistan; b Department Chemie, Fakultät für Naturwissenschaften, Universität Paderborn, Warburgerstrasse 100, D-33098 Paderborn, Germany

## Abstract

In the title mol­ecule, C_17_H_19_NO_5_, the amide plane is oriented at an angle of 41.5 (3)° with respect to the 2-methoxy­benzene ring. The three meth­oxy groups lie almost in the plane of the aromatic rings to which they are attached [C—O—C—C torsion angles of of 0.7 (4), −13.4 (4) and 3.1 (4)°], whereas the meth­oxy group at the 4-position of the 3,4,5-trimethoxy­benzene ring is nearly perpendicularly oriented [C—O—C—C torsion angle of 103.9 (3)°]. In the crystal structure, inter­molecular N—H⋯O hydrogen bonds link the mol­ecules into chains along [001].

## Related literature

The background of this work has been described in our earlier paper (Saeed *et al.* 2008 ▶). For a related structure, see: Parra *et al.* (2001 ▶).
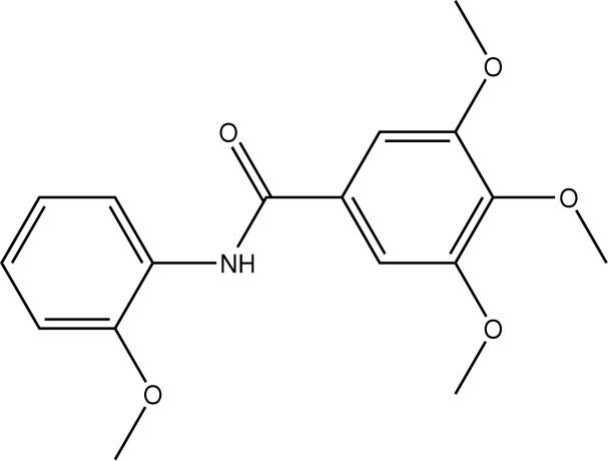


## Experimental

### 

#### Crystal data

C_17_H_19_NO_5_
*M* *_r_* = 317.33
Orthorhombic, 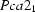

*a* = 7.409 (2) Å
*b* = 22.522 (6) Å
*c* = 9.681 (3) Å
*V* = 1615.4 (7) Å^3^
*Z* = 4
Mo *K*α radiation
μ = 0.10 mm^−1^
*T* = 120 K
0.50 × 0.44 × 0.20 mm

#### Data collection

Bruker SMART APEX diffractometer
Absorption correction: multi-scan (*SADABS*; Sheldrick, 2004 ▶) *T*_min_ = 0.953, *T*_max_ = 0.981
13253 measured reflections
2050 independent reflections
1902 reflections with *I* > 2σ(*I*)
*R*_int_ = 0.047

#### Refinement

*R*[*F* ^2^ > 2σ(*F* ^2^)] = 0.051
*wR*(*F* ^2^) = 0.126
*S* = 1.13
2050 reflections
216 parameters
2 restraints
H atoms treated by a mixture of independent and constrained refinement
Δρ_max_ = 0.37 e Å^−3^
Δρ_min_ = −0.20 e Å^−3^

### 

Data collection: *SMART* (Bruker, 2002 ▶); cell refinement: *SAINT* (Bruker, 2002 ▶); data reduction: *SAINT*; program(s) used to solve structure: *SHELXS97* (Sheldrick, 2008 ▶); program(s) used to refine structure: *SHELXL97* (Sheldrick, 2008 ▶); molecular graphics: *SHELXTL* (Sheldrick, 2008 ▶); software used to prepare material for publication: *SHELXTL*.

## Supplementary Material

Crystal structure: contains datablocks I, global. DOI: 10.1107/S1600536809027974/wm2245sup1.cif

Structure factors: contains datablocks I. DOI: 10.1107/S1600536809027974/wm2245Isup2.hkl

Additional supplementary materials:  crystallographic information; 3D view; checkCIF report

## Figures and Tables

**Table 1:** Hydrogen-bond geometry (Å, °)

*D*—H⋯*A*	*D*—H	H⋯*A*	*D*⋯*A*	*D*—H⋯*A*
N1—H1⋯O1^i^	0.895 (10)	2.182 (14)	3.066 (4)	169 (4)

Symmetry code: (i) 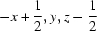.

**Fig. 1.**
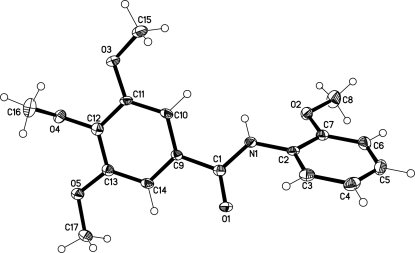
Molecular structure of title compound. Displacement ellipsoids are drawn at the 50% probability level.

**Fig. 2.**
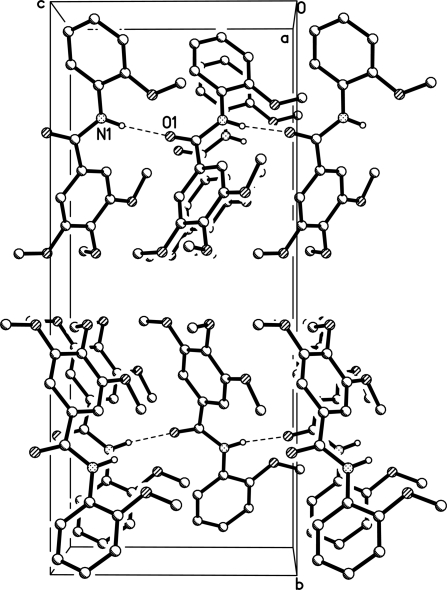
Crystal packing viewed along [100] with intermolecular hydrogen bonding pattern indicated as dashed lines. H-atoms not involved in hydrogen bonding are omitted.

## supplementary crystallographic information

### Comment

The background of this work has been described in our earlier paper (Saeed *et al.* 2008).

The molecular structure of the title compound (Fig. 1) is similar to that of ICULOH (Parra *et al.*, 2001), but with 3,4,5-methoxy substitution of the benzamide ring. Methoxy groups O2, O3 and O5 lie almost in plane of the corresponding aromatic rings with torsion angles C8–O2–C7–C6 of 0.7 (4)°, C15–O3–C11–C10 of -13.4 (4)° and C17–O5–C13–C14 of 3.1 (4)°, respectively, whereas the O4-group is nearly perpendicular oriented with C16–O4–C12–C13 of 103.9 (3)°. The two aromatic planes make a dihedral angle of 67.66 (9)° and the angle between the amide group and the 2-methoxy benzene ring is 41.5 (3)°. In the cystal structure, intermolecular N–H···O hydrogen bonds (Table 1) link the molecules into infinite chains along the [001] direction (Fig. 2).

### Experimental

3,4,5-Trimethoxybenzoyl chloride (1 mmol) in CHCl_3_ was treated with 2-methoxyaniline (3.5 mmol) under a nitrogen atmosphere at reflux conditions for 5 h. Upon cooling, the reaction mixture was diluted with CHCl_3_ and washed consecutively with 1 *M* aq HCl and saturated aq NaHCO_3_. The organic layer was dried over anhydrous sodium sulfate and concentrated under reduced pressure. Crystallization of the residue in methanol afforded the title compound (84%) as white needles: Anal. calc. for C_17_H_19_NO_5_: C 64.34, H 6.03, N 4.41%; found: C 64.31, H 6.09, N 4.34%

### Refinement

All H atoms were clearly identified in difference syntheses, then refined at calculated positions riding on the carbon atoms (C–H = 0.95–0.99 Å) with isotropic displacement parameters *U*_iso_(H) = 1.2U(Ceq) or 1.5U(–CH_3_). All CH_3_ hydrogen atoms were allowed to rotate but not to tip. H(N) was refined freely with a restained (*DFIX*) N–H distance. The title compound crystallizes in the non-centrosymmetric space group P ca21; however, in the absence of significant anomalous scattering effects, the Flack parameter is essentially meaningless. Accordingly, Friedel pairs were merged.

### Crystal data

**Table:**

C_17_H_19_NO_5_	*F*(000) = 672
*M**_r_* = 317.33	*D*_x_ = 1.305 Mg m^−^^3^
Orthorhombic, *P**c**a*2_1_	Mo *K*α radiation, λ = 0.71073 Å
Hall symbol: P 2c -2ac	Cell parameters from 887 reflections
*a* = 7.409 (2) Å	θ = 2.9–27.6°
*b* = 22.522 (6) Å	µ = 0.10 mm^−^^1^
*c* = 9.681 (3) Å	*T* = 120 K
*V* = 1615.4 (7) Å^3^	Prism, colourless
*Z* = 4	0.50 × 0.44 × 0.20 mm

### Data collection

**Table:**

Bruker SMART APEX diffractometer	2050 independent reflections
Radiation source: sealed tube	1902 reflections with *I* > 2σ(*I*)
graphite	*R*_int_ = 0.047
φ and ω scans	θ_max_ = 27.9°, θ_min_ = 1.8°
Absorption correction: multi-scan (*SADABS*; Sheldrick, 2004)	*h* = −9→9
*T*_min_ = 0.953, *T*_max_ = 0.981	*k* = −29→25
13253 measured reflections	*l* = −12→12

### Refinement

**Table:**

Refinement on *F*^2^	Primary atom site location: structure-invariant direct methods
Least-squares matrix: full	Secondary atom site location: difference Fourier map
*R*[*F*^2^ > 2σ(*F*^2^)] = 0.051	Hydrogen site location: difference Fourier map
*wR*(*F*^2^) = 0.126	H atoms treated by a mixture of independent and constrained refinement
*S* = 1.13	*w* = 1/[σ^2^(*F*_o_^2^) + (0.0648*P*)^2^ + 0.6933*P*] where *P* = (*F*_o_^2^ + 2*F*_c_^2^)/3
2050 reflections	(Δ/σ)_max_ < 0.001
216 parameters	Δρ_max_ = 0.37 e Å^−^^3^
2 restraints	Δρ_min_ = −0.20 e Å^−^^3^

### Special details

**Table:**

Geometry. All e.s.d.'s (except the e.s.d. in the dihedral angle between two l.s. planes) are estimated using the full covariance matrix. The cell e.s.d.'s are taken into account individually in the estimation of e.s.d.'s in distances, angles and torsion angles; correlations between e.s.d.'s in cell parameters are only used when they are defined by crystal symmetry. An approximate (isotropic) treatment of cell e.s.d.'s is used for estimating e.s.d.'s involving l.s. planes.
Refinement. Refinement of *F*^2^ against ALL reflections. The weighted *R*-factor *wR* and goodness of fit *S* are based on *F*^2^, conventional *R*-factors *R* are based on *F*, with *F* set to zero for negative *F*^2^. The threshold expression of *F*^2^ > σ(*F*^2^) is used only for calculating *R*-factors(gt) *etc*. and is not relevant to the choice of reflections for refinement. *R*-factors based on *F*^2^ are statistically about twice as large as those based on *F*, and *R*- factors based on ALL data will be even larger.

### Fractional atomic coordinates and isotropic or equivalent isotropic displacement parameters (Å^2^)

**Table:**

	*x*	*y*	*z*	*U*_iso_*/*U*_eq_	
O1	0.1314 (3)	0.22502 (9)	1.0268 (2)	0.0232 (5)	
O2	−0.0063 (3)	0.16331 (10)	0.5858 (2)	0.0246 (5)	
O3	0.7319 (3)	0.33720 (9)	0.7087 (2)	0.0258 (5)	
O4	0.6652 (3)	0.43489 (9)	0.8620 (3)	0.0246 (5)	
O5	0.4036 (3)	0.43643 (9)	1.0503 (3)	0.0263 (5)	
N1	0.1909 (3)	0.19743 (11)	0.8055 (3)	0.0199 (5)	
H1	0.232 (7)	0.2095 (18)	0.723 (3)	0.053 (13)*	
C1	0.2067 (3)	0.23480 (13)	0.9166 (3)	0.0186 (6)	
C2	0.0968 (3)	0.14253 (13)	0.8110 (3)	0.0189 (6)	
C3	0.1076 (4)	0.10537 (14)	0.9251 (4)	0.0242 (6)	
H3A	0.1804	0.1163	1.0018	0.029*	
C4	0.0117 (4)	0.05177 (14)	0.9278 (4)	0.0285 (7)	
H4A	0.0179	0.0268	1.0068	0.034*	
C5	−0.0923 (4)	0.03511 (14)	0.8150 (4)	0.0294 (7)	
H5A	−0.1575	−0.0012	0.8169	0.035*	
C6	−0.1012 (4)	0.07152 (14)	0.6989 (4)	0.0270 (7)	
H6A	−0.1718	0.0598	0.6217	0.032*	
C7	−0.0069 (4)	0.12518 (13)	0.6956 (3)	0.0211 (6)	
C8	−0.1114 (5)	0.14694 (18)	0.4675 (4)	0.0382 (9)	
H8A	−0.0681	0.1089	0.4312	0.057*	
H8B	−0.0996	0.1776	0.3961	0.057*	
H8C	−0.2385	0.1432	0.4942	0.057*	
C9	0.3260 (4)	0.28794 (12)	0.8974 (3)	0.0167 (5)	
C10	0.4689 (4)	0.28683 (13)	0.8036 (3)	0.0183 (5)	
H10A	0.4859	0.2532	0.7457	0.022*	
C11	0.5872 (4)	0.33511 (13)	0.7945 (3)	0.0181 (5)	
C12	0.5583 (4)	0.38548 (13)	0.8771 (3)	0.0204 (6)	
C13	0.4143 (4)	0.38603 (12)	0.9724 (3)	0.0194 (6)	
C14	0.2986 (4)	0.33723 (12)	0.9835 (3)	0.0190 (6)	
H14A	0.2026	0.3374	1.0486	0.023*	
C15	0.7858 (5)	0.28235 (14)	0.6464 (4)	0.0328 (8)	
H15A	0.7979	0.2518	0.7179	0.049*	
H15B	0.9019	0.2877	0.5995	0.049*	
H15C	0.6945	0.2700	0.5790	0.049*	
C16	0.8392 (5)	0.42964 (16)	0.9272 (5)	0.0367 (9)	
H16A	0.8234	0.4255	1.0273	0.055*	
H16B	0.9109	0.4652	0.9077	0.055*	
H16C	0.9018	0.3946	0.8911	0.055*	
C17	0.2685 (5)	0.43817 (14)	1.1558 (4)	0.0323 (7)	
H17A	0.1487	0.4352	1.1135	0.048*	
H17B	0.2779	0.4757	1.2067	0.048*	
H17C	0.2865	0.4049	1.2195	0.048*	

### Atomic displacement parameters (Å^2^)

**Table:**

	*U*^11^	*U*^22^	*U*^33^	*U*^12^	*U*^13^	*U*^23^
O1	0.0229 (10)	0.0307 (11)	0.0159 (10)	−0.0039 (8)	0.0057 (9)	−0.0036 (9)
O2	0.0247 (11)	0.0311 (11)	0.0179 (10)	−0.0079 (9)	−0.0033 (8)	−0.0018 (9)
O3	0.0239 (10)	0.0295 (10)	0.0242 (12)	−0.0046 (9)	0.0100 (10)	−0.0015 (9)
O4	0.0241 (10)	0.0229 (11)	0.0268 (11)	−0.0044 (8)	0.0023 (9)	0.0033 (9)
O5	0.0285 (11)	0.0234 (10)	0.0270 (13)	−0.0027 (8)	0.0081 (10)	−0.0053 (9)
N1	0.0205 (11)	0.0271 (12)	0.0121 (11)	−0.0043 (9)	0.0007 (10)	−0.0019 (10)
C1	0.0142 (12)	0.0266 (14)	0.0150 (13)	0.0016 (10)	−0.0024 (10)	−0.0014 (11)
C2	0.0150 (12)	0.0240 (14)	0.0176 (14)	0.0001 (10)	0.0058 (11)	−0.0033 (12)
C3	0.0207 (14)	0.0289 (15)	0.0231 (16)	0.0044 (11)	0.0028 (12)	−0.0002 (12)
C4	0.0266 (15)	0.0269 (15)	0.0320 (18)	0.0057 (12)	0.0090 (14)	0.0062 (14)
C5	0.0242 (14)	0.0203 (14)	0.044 (2)	−0.0035 (11)	0.0069 (15)	−0.0030 (14)
C6	0.0223 (14)	0.0281 (15)	0.0307 (18)	−0.0025 (12)	0.0014 (13)	−0.0079 (13)
C7	0.0180 (13)	0.0242 (14)	0.0211 (15)	0.0006 (11)	0.0042 (11)	−0.0033 (12)
C8	0.0381 (19)	0.049 (2)	0.0276 (18)	−0.0158 (16)	−0.0144 (16)	0.0038 (17)
C9	0.0160 (12)	0.0243 (13)	0.0098 (12)	−0.0004 (10)	−0.0027 (10)	−0.0004 (10)
C10	0.0207 (12)	0.0245 (13)	0.0098 (12)	0.0018 (10)	−0.0005 (11)	−0.0018 (11)
C11	0.0163 (11)	0.0272 (14)	0.0107 (13)	0.0006 (10)	0.0026 (10)	0.0014 (11)
C12	0.0223 (13)	0.0218 (14)	0.0171 (14)	0.0000 (11)	−0.0036 (11)	0.0038 (11)
C13	0.0197 (13)	0.0228 (14)	0.0156 (14)	0.0015 (10)	−0.0031 (11)	0.0001 (12)
C14	0.0178 (12)	0.0275 (14)	0.0118 (12)	0.0025 (10)	−0.0008 (11)	−0.0007 (11)
C15	0.0301 (17)	0.0315 (15)	0.0368 (19)	−0.0044 (12)	0.0173 (16)	−0.0034 (16)
C16	0.0308 (17)	0.0338 (19)	0.045 (2)	−0.0093 (14)	−0.0069 (17)	0.0000 (16)
C17	0.0352 (17)	0.0316 (15)	0.0300 (18)	−0.0030 (14)	0.0107 (16)	−0.0127 (15)

### Geometric parameters (Å, °)

**Table:**

O1—C1	1.224 (4)	C6—H6A	0.9500
O2—C7	1.366 (4)	C8—H8A	0.9800
O2—C8	1.434 (4)	C8—H8B	0.9800
O3—C11	1.357 (3)	C8—H8C	0.9800
O3—C15	1.432 (4)	C9—C10	1.395 (4)
O4—C12	1.374 (4)	C9—C14	1.403 (4)
O4—C16	1.441 (4)	C10—C11	1.400 (4)
O5—C13	1.365 (4)	C10—H10A	0.9500
O5—C17	1.431 (4)	C11—C12	1.404 (4)
N1—C1	1.371 (4)	C12—C13	1.410 (4)
N1—C2	1.420 (4)	C13—C14	1.398 (4)
N1—H1	0.895 (10)	C14—H14A	0.9500
C1—C9	1.500 (4)	C15—H15A	0.9800
C2—C3	1.388 (4)	C15—H15B	0.9800
C2—C7	1.412 (4)	C15—H15C	0.9800
C3—C4	1.401 (5)	C16—H16A	0.9800
C3—H3A	0.9500	C16—H16B	0.9800
C4—C5	1.389 (5)	C16—H16C	0.9800
C4—H4A	0.9500	C17—H17A	0.9800
C5—C6	1.392 (5)	C17—H17B	0.9800
C5—H5A	0.9500	C17—H17C	0.9800
C6—C7	1.396 (4)		
			
C7—O2—C8	117.3 (2)	C10—C9—C1	120.9 (2)
C11—O3—C15	116.7 (2)	C14—C9—C1	118.2 (2)
C12—O4—C16	113.8 (3)	C9—C10—C11	120.1 (3)
C13—O5—C17	117.3 (2)	C9—C10—H10A	119.9
C1—N1—C2	123.1 (2)	C11—C10—H10A	119.9
C1—N1—H1	119 (3)	O3—C11—C10	124.1 (3)
C2—N1—H1	118 (3)	O3—C11—C12	116.2 (2)
O1—C1—N1	122.3 (3)	C10—C11—C12	119.8 (3)
O1—C1—C9	121.4 (3)	O4—C12—C11	120.4 (3)
N1—C1—C9	116.3 (2)	O4—C12—C13	119.9 (3)
C3—C2—C7	119.7 (3)	C11—C12—C13	119.7 (3)
C3—C2—N1	121.8 (3)	O5—C13—C14	125.1 (3)
C7—C2—N1	118.6 (3)	O5—C13—C12	114.4 (2)
C2—C3—C4	120.3 (3)	C14—C13—C12	120.5 (3)
C2—C3—H3A	119.8	C13—C14—C9	119.1 (3)
C4—C3—H3A	119.8	C13—C14—H14A	120.4
C5—C4—C3	120.0 (3)	C9—C14—H14A	120.4
C5—C4—H4A	120.0	O3—C15—H15A	109.5
C3—C4—H4A	120.0	O3—C15—H15B	109.5
C4—C5—C6	120.1 (3)	H15A—C15—H15B	109.5
C4—C5—H5A	119.9	O3—C15—H15C	109.5
C6—C5—H5A	119.9	H15A—C15—H15C	109.5
C5—C6—C7	120.3 (3)	H15B—C15—H15C	109.5
C5—C6—H6A	119.8	O4—C16—H16A	109.5
C7—C6—H6A	119.8	O4—C16—H16B	109.5
O2—C7—C6	124.3 (3)	H16A—C16—H16B	109.5
O2—C7—C2	116.1 (2)	O4—C16—H16C	109.5
C6—C7—C2	119.6 (3)	H16A—C16—H16C	109.5
O2—C8—H8A	109.5	H16B—C16—H16C	109.5
O2—C8—H8B	109.5	O5—C17—H17A	109.5
H8A—C8—H8B	109.5	O5—C17—H17B	109.5
O2—C8—H8C	109.5	H17A—C17—H17B	109.5
H8A—C8—H8C	109.5	O5—C17—H17C	109.5
H8B—C8—H8C	109.5	H17A—C17—H17C	109.5
C10—C9—C14	120.7 (3)	H17B—C17—H17C	109.5
			
C2—N1—C1—O1	−4.4 (4)	C1—C9—C10—C11	−175.1 (3)
C2—N1—C1—C9	174.0 (2)	C15—O3—C11—C10	−13.4 (4)
C1—N1—C2—C3	−39.8 (4)	C15—O3—C11—C12	167.1 (3)
C1—N1—C2—C7	141.6 (3)	C9—C10—C11—O3	178.4 (3)
C7—C2—C3—C4	−2.0 (4)	C9—C10—C11—C12	−2.0 (4)
N1—C2—C3—C4	179.4 (3)	C16—O4—C12—C11	−78.5 (4)
C2—C3—C4—C5	1.1 (5)	C16—O4—C12—C13	103.9 (3)
C3—C4—C5—C6	0.1 (5)	O3—C11—C12—O4	4.5 (4)
C4—C5—C6—C7	−0.5 (5)	C10—C11—C12—O4	−175.1 (3)
C8—O2—C7—C6	0.7 (4)	O3—C11—C12—C13	−177.9 (3)
C8—O2—C7—C2	−179.6 (3)	C10—C11—C12—C13	2.5 (4)
C5—C6—C7—O2	179.2 (3)	C17—O5—C13—C14	3.1 (4)
C5—C6—C7—C2	−0.4 (4)	C17—O5—C13—C12	−175.6 (3)
C3—C2—C7—O2	−178.0 (2)	O4—C12—C13—O5	−4.6 (4)
N1—C2—C7—O2	0.6 (4)	C11—C12—C13—O5	177.7 (3)
C3—C2—C7—C6	1.7 (4)	O4—C12—C13—C14	176.6 (3)
N1—C2—C7—C6	−179.7 (3)	C11—C12—C13—C14	−1.1 (4)
O1—C1—C9—C10	151.4 (3)	O5—C13—C14—C9	−179.5 (3)
N1—C1—C9—C10	−27.1 (4)	C12—C13—C14—C9	−0.8 (4)
O1—C1—C9—C14	−24.0 (4)	C10—C9—C14—C13	1.3 (4)
N1—C1—C9—C14	157.6 (2)	C1—C9—C14—C13	176.7 (2)
C14—C9—C10—C11	0.1 (4)		

### Hydrogen-bond geometry (Å, °)

**Table:**

*D*—H···*A*	*D*—H	H···*A*	*D*···*A*	*D*—H···*A*
N1—H1···O1^i^	0.90 (1)	2.18 (1)	3.066 (4)	169 (4)

Symmetry codes: (i) −*x*+1/2, *y*, *z*−1/2.
